# The Mediating Role of Basic Psychological Needs and Meaning in Life in Adolescent Suicidal Ideation

**DOI:** 10.3390/bs15010014

**Published:** 2024-12-27

**Authors:** Jiayi Li, Jinqian Liao, Shuai Chen, Cheng Guo

**Affiliations:** 1 Research Center of Mental Health Education, Faculty of Psychology, Southwest University, Chongqing 400715, China; lijiayi67@email.swu.edu.cn (J.L.); 1155171960@link.cuhk.edu.hk (J.L.); 2 School of Psychology & Center for Studies of Psychological Application, South China Normal University, Guangzhou 510631, China; chenshuai@m.scnu.edu.cn

**Keywords:** parental psychological control, suicidal ideation, middle school students, meaning in life, basic psychological needs

## Abstract

Suicidal ideation and behavior are critical psychological crises among children and adolescents, posing significant concerns for their mental health and safety. This cross-sectional study investigated the factors and underlying psychological mechanisms of suicidal ideation in adolescents. A total of 6474 middle school students from Sichuan and Hebei provinces, China, participated in the study. Data were collected using the Self-Assessment of Suicidal Ideation Scale, the Parental Psychological Control Scale, the Meaning in Life Scale, and the Basic Psychological Needs Scale. Statistical analyses, including mediation and chain mediation analyses, were conducted to examine the relationships between parental psychological control, basic psychological needs, meaning in life, and suicidal ideation. The results revealed the following: (1) Parental psychological control positively predicted suicidal ideation in middle school students; (2) basic psychological needs mediated the relationship between parental psychological control and suicidal ideation; (3) meaning in life mediated the relationship between parental psychological control and suicidal ideation; (4) basic psychological needs and meaning in life together played a chain-mediating role in this relationship. These findings highlight the importance of addressing parental psychological control and fostering a supportive family environment to meet adolescents’ psychological needs, enhance their sense of life meaning, and reduce suicidal ideation. Insights from this study provide valuable guidance for prevention and intervention strategies aimed at safeguarding adolescents’ mental health and well-being.

## 1. Introduction

Suicidal ideation and behavior are critical concerns for children and adolescents experiencing severe psychological distress, particularly among those subjected to adverse experiences such as bullying (Louise, 2017). Research indicates that approximately 10.86% to 30.5% of adolescents in Europe have admitted to having thoughts of ending their own lives (Kokkevi et al., 2012). Moreover, suicide has become the second-leading cause of death among adolescents aged 15 to 19 in China (Pan et al., 2021). Suicidal ideation, a precursor to suicidal behavior, is a key risk factor for both suicide attempts and completed suicide. Surveys indicate that approximately 85% of individuals who die by suicide have expressed suicidal ideation (Angelakis et al., 2020).

Suicidal ideation is a key risk factor for both suicide attempts and suicidal behavior, significantly predicting the occurrence of suicide (Darvishi et al., 2015). Therefore, the timely and accurate assessment of suicidal ideation, along with a thorough understanding of its influencing factors, is crucial to preventing and intervening in suicidal behavior.

Parental psychological control is a negative parenting style characterized by inducing guilt, withdrawing love, and asserting authority (Barber, 1996). When parents attempt to impose their views on their children in a subtle yet coercive manner, they fail to respond to the children’s emotional needs (Soenens & Vansteenkiste, 2010). This lack of emotional connection can cause psychological distress, particularly in adolescence, and may exacerbate suicidal ideation and behavior (Pinquart, 2017). Although scholars have recognized the association between high-control parenting and suicidal ideation in adolescents, limited research has explored the underlying mechanisms (Crockenberg, 1980). Based on the self-determination theory (Chirkov et al., 2003), Ecosystem theory (Crockenberg, 1980), and the Social Ecology Diathesis-Stress Model (Beckmann et al., 2021), this study investigates the relationship between parental psychological control and suicidal ideation in middle school students. Specifically, parental psychological control is conceptualized as the independent variable, while suicidal ideation serves as the dependent variable, while suicidal ideation serves as the dependent variable. This study further incorporates basic psychological needs and meaning in life as mediating variables to construct a chain mediation model. This approach aims to explore the key influencing factors and underlying psychological mechanisms contributing to suicidal ideation among middle school students. In this study, “middle school students” refers to adolescents in grades 7 to 12, typically aged 12 to 19 years. This research seeks to enrich theoretical frameworks and provide evidence-based support for suicide crisis interventions among adolescents.

### 1.1. Parental Psychological Control and Suicidal Ideation

Ecosystem theory suggests that individual development results from the interaction between individuals and their surrounding environment. The family is a critical microsystem in adolescent development, with parenting styles being a key mechanism for families to exert influence on individuals (Murray et al., 2012). Parental psychological control refers to a negative parenting behavior where parents use strategies such as emotional neglect, guilt induction, and love withdrawal to invade the children’s psychological world, suppress their autonomy, and force compliance (Craske et al., 2019). Parental psychological control refers to parenting behavior that is manipulative and intrudes into children’s psychological world (Cai et al., 2022). Previous studies have explored the underlying mechanisms of parental psychological control with individual internalizing problems. For example, parental psychological control can undermine the quality of the parent–child relationship, diminish the individual’s sense of control, increase dependency, foster negative self-schemas, and lead to difficulties in emotional regulation (Bilsen, 2018; Caldwell et al., 2019; Elbogen et al., 2021), thereby resulting in various internalizing problems. These internalizing problems—such as depression, anxiety, loneliness, and low self-esteem (Dworkin et al., 2022; Halgunseth et al., 2006; Keefe et al., 2004), are also known risk factors for suicidal ideation (Casas-Munoz et al., 2024).

The Social Ecology Diathesis-Stress Model highlights that stressful life events, such as negative parenting styles, can activate an individual’s cognitive vulnerability, leading to the adoption of negative cognitive schemas or belief systems in interpreting themselves and the external environment, ultimately leading to negative adaptive outcomes (Murray et al., 2012). Parental psychological control, a negative parenting style that invades and disrupts adolescent autonomy, is often viewed as a significant stressor that undermines the parent–child relationship (Barber, 1996). Prolonged parental psychological control can activate cognitive vulnerabilities and poor coping strategies in adolescents, resulting in various adjustment problems and contributing to the onset of suicidal ideation (Barber et al., 2005). Numerous studies have emphasized the significant correlations between parental psychological control and suicidal behavior (Deci et al., 2017). For example, Chinese adolescents who attempted suicide reported higher levels of perceived psychological control (Li et al., 2023). Compared with behavioral control, psychological control is a more significant family predictor of suicidal behavior in adolescents (Deci & Ryan, 2008). Therefore, based on the above theories and empirical studies, this study proposes the following:

**Hypothesis** **1.** *Parental psychological control positively predicts suicidal ideation in middle school students.*

### 1.2. Mediating Role of Basic Psychological Needs

Parental psychological control directly influences the emergence of suicidal ideation in middle school students while also indirectly affecting suicidal ideation through the individual’s internal psychological characteristics. In this indirect process, the satisfaction with the basic psychological needs of middle school students is an important psychological variable (Salway et al., 2019). Basic psychological needs are fundamental and innate requirements essential for healthy psychological development and well-being. According to the self-determination theory, these needs are considered “psychological nutrients” that support positive psychological growth. The theory identifies three core basic psychological needs: competence, relatedness, and autonomy (Deci et al., 2017). Competence refers to an individual’s ability to effectively handle tasks and challenges, while relatedness involves feeling connected to and supported by others. Autonomy, on the other hand, pertains to having control over one’s own actions and experiencing psychological freedom (Deci & Ryan, 2008). When these needs are not adequately met due to personal or external factors, it can have detrimental effects on mental health, potentially increasing the risk of suicidal ideation (Salway et al., 2019). Research indicates that individuals experiencing suicidal thoughts or behaviors often report unmet basic psychological needs (Van den Broeck et al., 2016). For instance, Ryan & Deci demonstrated a strong link between the frustration of basic psychological needs and suicidal ideation, suicidal behavior, and exposure to negative life events (Ryan & Deci, 2000). Stressors such as parental psychological control, which undermine the parent–child relationship, can significantly hinder the satisfaction of these needs (Hoeve et al., 2009). Moreover, basic psychological needs frequently act as mediating variables in the relationship between stressors and psychological outcomes, further underscoring their critical role in mental health.

Parental psychological control, as a poor parenting style, directly leads to the deterioration of the parent–child relationship and fails to satisfy the children’s need for relatedness among the basic psychological needs (Steger et al., 2006). Additionally, parental psychological control, through suppression and intrusion, stifles the child’s independent expression and autonomy, making it difficult for them to develop independent expression and autonomy, thereby failing to meet the child’s basic psychological needs (Hinduja & Patchin, 2019). Adolescents who do not satisfy basic psychological needs may experience feelings of social rejection and emotional burden, both of which are widely recognized risk factors for suicide in various theories of suicide. Casas’ research indicates that warm and close parent–child relationships can promote the development of adolescents’ internal resources, such as the fulfillment of basic psychological needs, and that positive parent–child relationships, through the mediating role of basic psychological needs, positively influence individuals’ subjective well-being (Ronnstad et al., 2018). Empirical studies have shown that inappropriate parental control hinders the satisfaction of adolescents’ basic psychological needs, thereby affecting their emotional health and behavioral adjustment (Deci et al., 2017). However, there still has been a lack of research directly examining the mediating role of basic psychological needs in the relationship between parental psychological control and suicidal ideation. Therefore, based on the above theories and empirical studies, this study proposes the following:

**Hypothesis** **2.** *Basic psychological needs play a mediating role in the relationship between parental psychological control and suicidal ideation in middle school students*.

### 1.3. Mediating Role of Meaning in Life

Meaning in life refers to an individual’s perception of their existence and its significance, encompassing their awareness of important aspects or relationships in life (Dowlati et al., 2010). Lack of meaning in life may lead to negative reactions such as withdrawal, hostility, or self-blame and can even result in suicidal ideation and behavior. Conversely, possessing meaning in life is an important protective factor against suicidal ideation (Casas-Munoz et al., 2024). A longitudinal study conducted on Spanish adolescents found a negative correlation between levels of meaning in life and the frequency of suicidal ideation (Wang et al., 2016). Additionally, research by Ronnstad et al. demonstrated that meaning in life negatively predicts suicidal ideation and that increasing meaning in life can reduce the levels of suicidal ideation (Ronnstad et al., 2018).

Based on the Ecosystem theory (Beckmann et al., 2021), the development of meaning in life in adolescents is also influenced by parental control, with parenting styles playing a crucial role. Empirical research indicates that adolescents’ meaning in life is related to parental supervision and authoritative parenting styles (Ryan & Deci, 2001). Parental supervision can help adolescents in developing social expectations and appropriate behaviors. Wang et al. conducted a longitudinal study examining the impact of parental behavioral control, psychological control, and the quality of the parent–child relationship on the development of meaning in life among adolescents in Hong Kong (Wang et al., 2016). The study found that parental behavioral control and the quality of the parent–child relationship positively predicted the initial levels of adolescents’ meaning in life, while initial levels of parental psychological control negatively predicted the starting state of meaning in life. Adolescents from families with higher-quality parent–child relationships reported significantly higher levels of meaning in life at each time point compared to those from families with poorer-quality relationships. Overall, parental behavioral control has a positive impact on adolescent development (Varese et al., 2012), while psychological control is negatively correlated with it.

Therefore, meaning in life is an important mediator in preventing adolescents from developing suicidal ideation, making it necessary to explore the mechanisms of the relationship between meaning in life, parental psychological control, and suicidal ideation. Based on the above theories and empirical findings, Hypothesis 3 is proposed as follows:

**Hypothesis** **3.** *Meaning in life plays a mediating role in the relationship between parental psychological control and suicidal ideation in middle school students.*

### 1.4. The Chain Mediating Role of Basic Psychological Needs and Meaning in Life

Moreover, basic psychological needs are closely related to meaning in life (Sjöström et al., 2004). The basic psychological needs satisfaction model suggests that when an individual’s basic psychological needs are satisfied, they are likely to experience a sense of meaning in life. Conversely, unsatisfied needs may lead to negative feelings such as self-doubt, insecurity, and self-rejection (Chen et al., 2015). Numerous studies have established significant correlations between different basic psychological needs and meaning in life. For example, Vansteenkiste et al. examined the relationship between three basic psychological needs and meaning in life and found that each basic need significantly predicted meaning in life (Vansteenkiste et al., 2020). Moreover, the satisfaction of each basic psychological need was shown to promote meaning in life through distinct pathways.

Meanwhile, the “Motivation-Life Meaning” model of suicidal ideation constructs a “motivation-meaning-of-life-suicidal ideation” relationship chain from a positive psychology orientation. The model suggests that a lack of meaning in life is the core cause of suicidal ideation, and a lack of meaning in life is caused by an increase in motivation due to adverse circumstances, such as the failure to meet basic psychological needs resulting in the depletion of the sense of meaning in life (Wang, 2013). Emotional belongingness is a crucial component of basic psychological needs and a significant source of meaning in life (Steger et al., 2006).

Parental psychological control, as a negative environmental factor, can diminish the fulfillment of these needs, increase motivational demands, and reduce meaning in life, thereby contributing to suicidal ideation (Choi & Kim, 2018). Therefore, basic psychological needs and meaning in life play a chain mediating role in the relationship between parental psychological control and suicidal ideation. In other words, excessive psychological control by parents prevents children from fulfilling their basic psychological needs, which in turn inhibits their sense of meaning in life, ultimately resulting in higher levels of suicidal ideation. Based on the above theories and empirical studies, Hypothesis 4 is proposed as follows:

**Hypothesis** **4.** *Basic psychological needs and meaning in life play a chain mediating role in the relationship between parental psychological control and suicidal ideation.*

## 2. Methods

### 2.1. Participants

This study employed a cross-sectional survey design to investigate the relationship between parental psychological control, basic psychological needs, meaning in life, and suicidal ideation among middle school students. Data were collected in October 2023 from 8342 students aged 12–19 years in Sichuan and Hebei provinces, China, using a convenience sampling method. This method was chosen due to logistical feasibility and the need to access a large and diverse sample quickly within the constraints of time and resources. Both male and female students were included. Inclusion criteria required participants to be currently enrolled in middle school during the study period, provide informed consent (from both students and their parents), and complete the questionnaire appropriately. Exclusion criteria included incomplete responses, evident response bias, or a masking factor score of 4 or higher on the suicidal ideation scale.

The survey was conducted in a classroom setting, with data collected online via the Questionnaire Star platform (Questionnaire Star, n.d.). a widely used online survey tool in China that enables the design, distribution, and collection of questionnaire-based data efficiently. Before completing the questionnaire, participants were provided with instructions explaining the purpose of the study, the structure of the questionnaire, and the confidentiality of their responses. They then completed the questionnaire independently within an allotted time of approximately 30 min.

This study was conducted according to the guidelines of the Declaration of Helsinki and approved Research Project Ethical Review Application Form, Faculty of Psychology, Southwest University of China (IRB protocol number: H23175 and approval date 15 September 2023). All participating students and their parents signed informed consent forms. Data collected through questionnaires were anonymized and stored securely to ensure confidentiality and compliance with data protection regulations.

### 2.2. Measures

#### 2.2.1. Parental Psychological Control

Parental psychological control was measured using the Parental Psychological Control Scale-Youth Version (PCS-YSR) developed (Choi & Kim, 2018), including ten items (e.g., “Dad/Mom is always trying to change my mind”). The questionnaire is scored on a 5-point scale, with 1 (very non-conforming) and 5 (very conforming), with higher scores indicating a higher level of perceived parental control. The total score for this scale ranges from 10 to 50. The Cronbach’s alpha coefficient for the questionnaire was 0.88. The scale demonstrated a good single-factor structure (χ^2^/*df* = 2.21, CFI = 0.94, TLI = 0.92, RMSEA = 0.06, SRMR = 0.04).

#### 2.2.2. Basic Psychological Needs

Basic psychological needs were measured using the simple version of the Basic Psychological Needs Scale developed by Weinstein and Ryan (2010), which assesses the extent to which individuals perceive their basic psychological needs as being fulfilled. The scale consists of 21 items divided into three dimensions: autonomy (7 items, e.g., “I feel that I can freely decide how to live my life”), competence (6 items, e.g., “I feel that I cannot handle many aspects of life well”), and relatedness (8 items, e.g., “I get along well with the people around me”). Participants responded on a 5-point Likert scale (1 = strongly disagree, 5 = strongly agree). Higher scores indicate greater satisfaction with basic psychological needs. The total score for this scale ranges from 21 to 105. This scale has also been used by Chinese researchers and has demonstrated good reliability and validity (Feng & Guo, 2017). In this study, the Cronbach’s alpha coefficient for the scale was 0.78. The scale demonstrated a good three-factor structure, namely autonomy, competence, and relatedness (χ^2^/*df* = 2.27, CFI = 0.92, TLI = 0.91, RMSEA = 0.07, SRMR = 0.05).

#### 2.2.3. Meaning in Life

The revised Chinese version of the Meaning in Life Scale was developed by Steger et al. (2006) and revised by Wang (2013). The scale consists of 10 items (e.g., “I understand the meaning of my life well”). Items covered include having meaning and seeking meaning. The items are scored on a 7-point scale, with 1 (very inconsistent) and 7 (completely consistent), and the mean score of the items is calculated, with higher scores indicating a stronger sense of meaning in life. The total score for this scale ranges from 10 to 70. In this study, the Cronbach’s alpha coefficient for the scale was 0.77. The scale demonstrated a good two-factor structure, namely the presence of meaning in life and the search for meaning (χ^2^/*df* = 2.01, CFI = 0.95, TLI = 0.93, RMSEA = 0.05, SRMR = 0.04).

#### 2.2.4. Suicidal Ideation

The Self-Assessment of Suicidal Ideation Scale (SASI) was developed by Xia and Wang (2002). The scale contains 26 items (e.g., “I feel that my life is a failure”) and covers factors including despair, optimism, sleep, and masking. The combined score of the despair, optimism, and sleep factors can be used as a total score for suicidal ideation. The total score for this scale ranges from 0 to 26. In this study, if the total score of suicidal ideation is 12 or more, and the score of the masking factor is less than 4, it can be concluded that suicidal ideation exists. The Cronbach’s alpha coefficient for this scale in this study was 0.81. The scale demonstrated a good four-factor structure, namely hopelessness, optimism, sleep, and masking (χ^2^/*df* = 2.23, CFI = 0.93, TLI = 0.91, RMSEA = 0.06, SRMR = 0.05).

### 2.3. Data Analysis Strategy

Descriptive statistical analyses were conducted using SPSS Statistics (version 27.0, IBM Corp, Armonk, NY, USA.) to summarize the sample characteristics, including means and standard deviations for quantitative variables (e.g., age, SES, parental psychological control, basic psychological needs, meaning in life, and suicidal ideation) and proportions for categorical variables (e.g., gender). Gender was coded as a dichotomous variable (female = 0, male = 1) to facilitate correlation and mediation analyses. Pearson correlation analyses were performed to examine the relationships between variables, as all were treated as continuous or dichotomous and met the assumptions for such analyses.

Structural equation modeling (SEM) and mediation analyses were conducted using Mplus 8.3 to test the hypothesized model. Model parameters were estimated using the Maximum Likelihood Method, and model fit was evaluated using multiple indices: Chi-square, CFI (≥0.90), TLI (≥0.90), RMSEA (≤0.08), and SRMR (≤0.08). A CFI and TLI above 0.95 indicate an excellent fit, while RMSEA values below 0.05 and SRMR values below 0.08 suggest a good fit. To test the mediation effects, the Bootstrap method (5000 resamples) was applied with 95% confidence intervals (CIs). Mediation effects were considered significant if the CI did not include zero. Statistical significance was set at *p* < 0.05 (Kline, 2018, p. 4).

## 3. Results

### 3.1. Descriptive Statistics and Correlation Analysis

**Figure 1:**
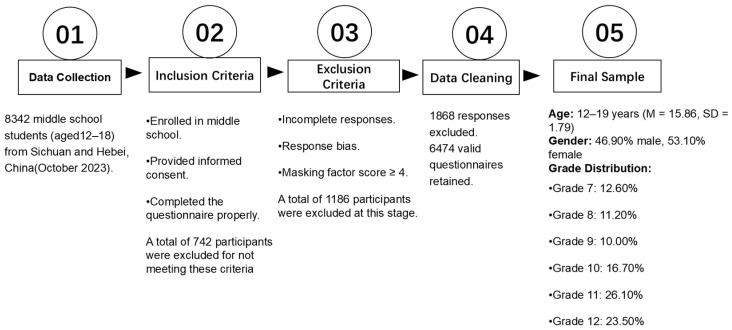
The flowchart shows the selection process of responders according to inclusion and exclusion criteria.

In Figure 1, after excluding 1868 questionnaires due to high masking factor scores or incomplete responses, 6474 valid questionnaires were retained, yielding an effective response rate of 77.61%. The participants’ ages ranged from 12 to 19 years (M = 15.86, SD = 1.79). A total of 6474 students participated, with the distribution by gender as follows: 3039 males (46.9%) and 3435 females (53.1%). The participants’ mean age was 15.86 years (SD = 1.79). The grade distribution of participants was as follows: 815 (12.6%) in Grade 7, 723 (11.2%) in Grade 8, 646 (10.0%) in Grade 9, 1078 (16.7%) in Grade 10, 1690 (26.1%) in Grade 11, and 1522 (23.5%) in Grade 12.

In this study, the overall detection rate of suicidal ideation among middle school students is 22.74%. Specifically, the detection rate is 20.89% for junior high school students and 23.66% for senior high school students.

Descriptive statistics and correlation analyses were performed on the variables, and the results are shown in Table 1. Parental psychological control, basic psychological needs, and meaning in life were significantly correlated with the suicidal ideation scores of the middle school students.

### 3.2. Hypothesis Testing

After controlling the age, gender, and family socioeconomic status (SES), a chained mediation model was constructed with parental psychological control as the independent variable, basic psychological needs and meaning in life as the mediating variables, and suicidal ideation as the dependent variable.

**Figure 2:**
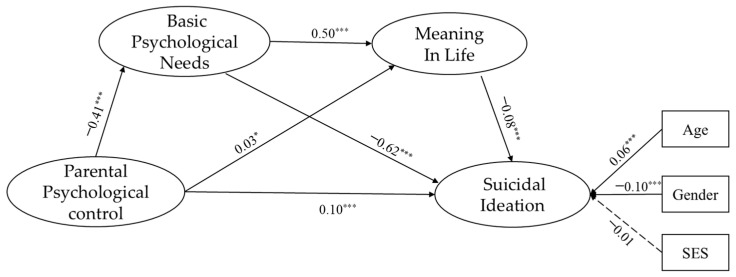
Basic psychological needs and meaning in life mediate the effect of parental psychological control on suicidal ideation. Note: All paths are standardized, dashed lines represent non-significant, and indicators for each latent variable are shown. * *p* < 0.05, *** *p* < 0.001.

The model fits the data excellently, as the results show in Figure 2 (*χ*^2^ = 734.029, *df* = 53, CFI = 0.980, TLI = 0.972, RMSEA = 0.045, SRMR = 0.032). These results collectively demonstrate that the model adequately represents the relationships among the study variables. Readers less familiar with these indices should note that they provide critical information about the model’s overall validity and its suitability for testing the hypothesized relationships.

Parental psychological control significantly positively predicted suicidal ideation (*β* = 0.10, *p* < 0.001) and negatively predicted basic psychological needs (*β* = −0.41, *p* < 0.001) but significantly positively predicted meaning in life (*β* = 0.03, *p* < 0.05). Basic psychological needs significantly positively predicted meaning in life (*β* = 0.50, *p* < 0.001) and significantly negatively predicted suicidal ideation (*β* = −0.62, *p* < 0.001). Meaning in life also significantly negatively predicted suicidal ideation (*β* = −0.08, *p* < 0.001).

Bootstrap mediation analyses in Table 2 reveal a significant separate mediating effect of basic psychological needs (*β* = 0.255, *SE* = 0.011, *p* < 0.001, 95% CI [0.237, 0.274]) accounting for 68.92% of the total effect. The separate mediating effect of the meaning of life was small but significant and in the opposite direction to what was expected (*β* = −0.002, *SE* = 0.001, *p* < 0.05, 95% CI [−0.004, −0.001]). The chain mediation between basic psychological needs and a sense of meaning in life is significant (*β* = 0.016, *SE* = 0.003, *p* < 0.001, 95% CI [0.012, 0.020]), accounting for 4.32% of the total effect.

## 4. Discussion

### 4.1. Predictors of Suicidal Ideation

This study aims to explore the relationship between parental psychological control and suicidal ideation among middle school students. The results indicate that parental psychological control significantly positively predicts suicidal ideation in adolescents, which is consistent with Hypothesis 1. Several studies have highlighted a significant correlation between parental psychological control and suicidal behaviors. For example, Chinese adolescents who have attempted suicide reported perceiving higher levels of parental psychological control (Pinquart, 2017). Furthermore, excessive parental control and the negative family atmosphere are significantly associated with adolescents’ suicidal ideation, and parental psychological control has a lasting impact on their children’s suicidal ideation (John et al., 2018). This conclusion is also consistent with the Ecosystem theory and the Social Ecology Diathesis-Stress Model. These theories emphasize the critical influence of family environment, particularly parenting styles, on adolescents’ psychological development (Keyes & Platt, 2024). Specifically, parental psychological control, as the negative parenting style, may induce internalizing problems in adolescents through mechanisms such as damaging parent–child relationships, diminishing feelings of control, enhancing dependency, fostering negative self-images, and leading to difficulties in emotional regulation (Scharf & Goldner, 2018). This, in turn, increases the risk of suicidal ideation. These findings are consistent with existing research that discusses the underlying mechanisms linking parental psychological control to individual internalizing problems (Anda et al., 2006), further confirming the negative impact of parental psychological control on adolescent mental health. This conclusion suggests that future crisis intervention work with adolescents should emphasize the adjustment in parenting styles.

### 4.2. The Mediating Role of Basic Psychological Needs

This study investigates the contribution of parental psychological control on suicidal ideation among middle school students, with a particular focus on the mediating role of basic psychological needs. The results indicate that basic psychological needs significantly negatively predict suicidal ideation, suggesting that the satisfaction of these needs plays a crucial role in reducing suicidal ideation among adolescents. When children’s basic psychological needs are fulfilled, they are more likely to develop positive self-identities and attitudes toward life, thereby lowering the risk of suicidal ideation (Choi & Kim, 2018).

Additionally, the findings reveal that parental psychological control not only directly positively predicts suicidal ideation but also indirectly affects suicidal ideation by influencing adolescents’ basic psychological needs. This discovery aligns with the self-determination theory, which posits that basic psychological needs serve as psychological nutrients vital for individual mental health. Specifically, parental psychological control, as a poor parenting style, can lead to the worsening of parent–child relationships, thereby failing to satisfy children’s relational needs, one of the basic psychological needs (Chirkov et al., 2003). This absence of a supportive relationship can leave children feeling lonely and misunderstood, which increases their risk of suicidal ideation (Ryan & Deci, 2000). Furthermore, parental psychological control can stifle children’s independent expression and autonomy, making it difficult for them to develop a healthy self-concept and perspective, thus failing to fulfill their autonomy needs (Soenens & Vansteenkiste, 2010). This deprivation of autonomy further undermines children’s self-identity and sense of control, exacerbating their suicidal thoughts. More importantly, such controlling behaviors can negatively impact children’s competence needs. Through excessive control and restriction, parents may hinder their children’s opportunities to explore and develop their potential, leading to feelings of helplessness and a lack of accomplishment. This frustration regarding competence needs can further diminish children’s self-worth and self-efficacy, exacerbate their internal despair and helplessness, and thus make them more vulnerable to suicidal ideation (Casas-Munoz et al., 2024).

Therefore, parental psychological control affects children’s basic psychological needs through multiple pathways, ultimately increasing the risk of suicidal thoughts. In summary, this study highlights the significant mediating role of basic psychological needs in the relationship between parental psychological control and suicidal ideation among middle school students. To mitigate the risk of suicidal ideation in adolescents, interventions should focus on improving parenting styles and enhancing the fulfillment of children’s basic psychological needs. Evidence-based programs such as the Positive Parenting Program (Triple P) or family-based interventions can provide structured approaches to fostering supportive parenting behaviors and meeting adolescents’ psychological needs. The Positive Parenting Program (Triple P) is a multi-level, evidence-based intervention designed to improve parenting practices, enhance parent–child relationships, and promote a positive family environment, which can reduce risk factors for adolescent mental health issues, including suicidal ideation. Similarly, family-based interventions focus on improving communication, strengthening emotional bonds, and addressing family conflicts to create a protective environment that supports adolescents’ psychological well-being and resilience. These programs help parents recognize and respond effectively to their children’s emotional needs, reducing stressors that might contribute to suicidal ideation.

### 4.3. The Mediating Role of Meaning in Life

This study investigates the mediating role of meaning in life in the relationship between parental psychological control and suicidal ideation in middle school students. The findings provide some support for Hypothesis 3 while also revealing complex interactions. Notably, one of the key findings indicates that parental psychological control significantly and positively predicts meaning in life, which is contrary to initial expectations. This may reflect a complex psychological process in which adolescents attempt to find meaning in life under conditions of psychological control. This may be due to the masking effect of basic psychological needs in the process of parental psychological control over the sense of meaning in life. In other words, although parental psychological control has a direct positive effect on the sense of meaning in life, this effect may be more indirect through the weakening of basic psychological needs. Individuals are more likely to experience a sense of meaning in life when basic psychological needs are met (Kohls et al., 2021). When these basic psychological needs—autonomy (e.g., the ability to act according to one’s own values and interests), competence (e.g., the sense of mastery and effectiveness in dealing with challenges), and relatedness (e.g., feeling understood and connected to others)—are denied, individuals may struggle to develop a genuine sense of meaning in life (Deci & Ryan, 2000). For instance, adolescents experiencing parental psychological control may feel restricted in their decision-making (autonomy), incapable of achieving their goals (competence), and isolated from meaningful relationships (relatedness). This deprivation of basic psychological needs may lead adolescents to seek meaning in life as an adaptive response to cope with adverse environments. However, this search for meaning may lack authenticity and fail to provide deep psychological fulfillment, as it stems from a compensatory mechanism rather than an intrinsic process.

The variability in these findings underscores the need for a more detailed exploration of individual differences and contextual factors (Milner et al., 2018). When individuals perceive support from their social networks—such as family, friends, and schools—they are more likely to develop positive attitudes and values, thereby attributing deeper meaning to their lives (Folkman & Lazarus, 1980). In contrast, if individuals experience rejection, isolation, or neglect in their social environments, they may feel that life lacks meaning, which can lead to suicidal ideation. Moreover, future studies should examine the variability in data, such as differences across demographic subgroups or cultural contexts, to provide a more nuanced understanding of this phenomenon.

In summary, when faced with parental psychological control, some adolescents may attempt to compensate for the lack of social support by seeking and establishing their sense of meaning in life. They may value their relationships with others more and work harder to pursue their personal goals and values as a way to enhance their sense of social belonging and meaning in life. Nevertheless, meaning in life remains a significant protective factor, significantly negatively predicting suicidal ideation (Miranda-Mendizabal et al., 2019). This finding aligns with previous research indicating that individuals with a high sense of meaning in life can effectively mitigate the negative impact of crisis events on mental health (Sjöström et al., 2004). Therefore, enhancing adolescents’ sense of meaning in life may be an effective strategy for preventing suicidal ideation.

### 4.4. The Chain Mediating Role of Basic Psychological Needs and Meaning in Life

Based on the “Motivation-Meaning in Life” model of suicidal ideation and the model of basic psychological needs satisfaction, this study explores the chain mediating role of basic psychological needs and meaning in life in the relationship between parental psychological control and suicidal ideation in middle school students. The results indicate that the mediating role of basic psychological needs is significant and accounts for a large proportion of the total effect. This suggests that basic psychological needs serve as an important mediator in the relationship between parental psychological control and suicidal ideation. In contrast, the separate mediating effect of meaning in life was small but significant and in the opposite direction of expectation, probably due to the masking effect of basic psychological needs.

However, the chain mediation effect of both basic psychological needs and meaning in life remains significant, indicating that both factors play a crucial role in the relationship between parental psychological control and suicidal ideation. Specifically, this suggests that parental psychological control not only directly affects the fulfillment of adolescents’ basic psychological needs but also indirectly impacts their sense of meaning in life by affecting basic psychological needs. When parental control leads to unmet basic psychological needs, adolescents are more likely to feel a lack of life meaning and value, thereby increasing their risk of suicidal ideation (Kline, 2018). The chain mediation effect reveals the complex mechanisms by which parental psychological control influences suicidal ideation in adolescents.

In summary, this study supports the chain-mediated role of basic psychological needs and meaning in life in the relationship between parental psychological control and suicidal ideation in middle school students. The findings provide new insights for developing future intervention programs focused on preventing suicidal ideation in middle school students. For instance, family-centered approaches could be effective by fostering open communication, reducing parental psychological control, and creating emotionally supportive environments. Additionally, interventions could emphasize strategies to enhance adolescents’ autonomy, competence, and sense of meaning in life, such as skill-building workshops, resilience training, and mentorship programs. These measures could complement existing frameworks to better address the psychological factors contributing to suicidal ideation.

### 4.5. Strengths, Limitations, and Future Direction

Building on the existing research, this study deeply explores the role of parental psychological control in the potential psychological processes underlying suicidal ideation in middle school students, offering both theoretical and practical value. Theoretically, this study reveals the complex interplay between parental psychological control, basic psychological needs, meaning in life, and suicidal ideation, which is crucial for understanding the mechanisms behind the formation and development of suicidal ideation in adolescents. Practically, investigating the psychological processes associated with suicidal ideation in middle school students is vital for prevention and intervention efforts. First, parents should be highly aware of their psychological control over their children, as excessive control can lead to poor parent–child relationships, which are a prerequisite for the development of suicidal ideation in adolescents. Second, enhancing adolescents’ fulfillment of basic psychological needs and the sense of meaning in life can help reduce the risk of suicidal ideation.

However, this study also has several limitations. Firstly, a cross-sectional research design was employed, which does not allow for the inference of causal relationships between the variables. Future research could consider a longitudinal design, as it would allow for tracking changes in variables over time, providing a clearer understanding of the directionality and potential causality of relationships. Second, self-reported questionnaires may introduce biases, such as social desirability bias and recall bias, leading to responses that may not accurately reflect participants’ true feelings. Additionally, many questionnaires were excluded due to incomplete or inconsistent responses, which could introduce a masking effect and limit the generalizability of the findings. For example, adolescents with higher psychological distress might have been less likely to complete the questionnaires, potentially underestimating the prevalence of suicidal ideation. Future research could address these issues by incorporating multiple data sources (e.g., parent or teacher reports), improving data collection methods, and using data imputation techniques to reduce the impact of missing or excluded data.

## 5. Conclusions, Limitations, and Prospective

This study confirms the mediating role of basic psychological needs in the relationship between parental psychological control and suicidal ideation and reveals a chain mediation mechanism involving both basic psychological needs and meaning in life. These findings deepen our understanding of how parental psychological control influences adolescents’ suicidal ideation and provide new insights into prevention and intervention strategies. However, this study has some limitations. First, the cross-sectional design limits causal inference, and future research should adopt longitudinal or experimental designs. Second, the reliance on self-reported data may introduce bias, and incorporating multi-informant or objective assessments could improve validity. Finally, the sample’s cultural and regional specificity may limit generalizability, warranting further studies in diverse contexts. Future research and practice should focus on enhancing adolescents’ basic psychological needs and sense of meaning in life to mitigate the negative effects of parental psychological control. Interventions could include parental education programs to promote healthier communication styles and adolescent-focused programs to foster resilience, autonomy, and purpose.

## Figures and Tables

**Table 1:** Descriptive statistics and correlation analysis (*N* = 6474).

Variables	*M*	*SD*	Skewness	Kurtosis	1	2	3	4	5	6	7
1. Age	15.86	1.79	−0.391	−0.725	1						
2. Gender	0.47	0.50	0.123	−1.985	−0.034 **	1					
3. SES	4.34	1.47	0.200	1.626	−0.085 ***	0.039 **	1				
4. Parental psychological control	2.28	0.84	0.486	0.007	0.000	0.054 ***	0.002	1			
5. Basic psychological needs	2.77	0.62	0.243	−0.351	−0.079 ***	0.006	0.108 ***	−0.350 ***	1		
6. Meaning in life	4.87	1.06	−0.539	1.338	0.018	0.002	0.077 ***	−0.156 ***	0.441 ***	1	
7. Suicidal ideation	6.98	5.34	0.579	−0.674	0.095 ***	−0.091 ***	−0.080 ***	0.308 ***	−0.582 ***	−0.346 ***	1

Note: gender (female = 0, male = 1), SES = socioeconomic status, ** *p* < 0.01, *** *p* < 0.001.

**Table 2:** Analysis of mediating effects of basic psychological needs and meaning in life.

Pathway	*β*	Boot SE	Bootstrap 95% CI		Percentage
			Min	Max	
Parental psychological control→suicidal ideation	0.101		0.078	0.124	27.30%
Parental psychological control→basic psychological needs →suicidal ideation	0.255	0.011	0.237	0.274	68.92%
Parental psychological control→meaning in life→suicidal ideation	−0.002	0.001	−0.004	−0.001	−0.54%
Parental psychological control→basic psychological needs→meaning in life→suicidal ideation	0.016	0.003	0.012	0.020	4.32%
Total indirect effect	0.269	0.011	0.252	0.287	72.70%

## Data Availability

Data are available from the corresponding author upon reasonable request.
